# National Trends in Total Ankle Replacement in Sweden: Demographic Shifts and Regional Disparities (2008-2023)

**DOI:** 10.1177/24730114261423178

**Published:** 2026-03-09

**Authors:** Michael Axenhus, Fatih Uludag, Viktor Mili-Schmidt

**Affiliations:** 1 Department of Orthopaedic Surgery, Danderyd Hospital, Stockholm, Sweden; 2 Department of Clinical Sciences at Danderyd Hospital, Karolinska Institutet, Stockholm, Sweden

**Keywords:** Ankle, foot, joint replacement

## Abstract

**Background:**

Total ankle replacement (TAR) is a surgical option for end-stage ankle arthritis, offering pain relief while preserving joint mobility. Although TAR utilization has increased globally, limited data exist regarding demographic and regional trends in Sweden in the last decade. This study aims to analyze national trends in TAR between 2008 and 2023, with a focus on age, sex, and geographic disparities.

**Methods:**

This retrospective cohort study used data from the Swedish National Patient Register (NPR) to identify all patients aged ≥15 years who underwent primary cementless TAR between January 1, 2008, and December 31, 2023. Exploratory analyses used descriptive statistics to examine trends in procedure volume, incidence, and demographic distribution. Regional incidence rates were calculated using population data.

**Results:**

A total of 1255 primary TAR procedures were identified during the study period. The volume of surgical cases increased by 205%, from 63 cases in 2008 to 129 cases in 2023. The incidence doubled from 0.6 to 1.2 per 100 000 inhabitants, despite a 14% increase in population size between 2008 and 2023. Female patients constituted 55% of overall cases, though male patients became the majority by 2023. Nearly half of all TARs were performed in individuals aged ≥65 years, with the 70-79-year age group showing the greatest relative increase (350%). Regional disparities were noted, with high variability in incidence between regions. Projections indicate that future incidence will rise for men and decrease for women.

**Conclusion:**

The utilization of TAR in Sweden has increased substantially over the past 16 years, with notable demographic shifts toward older and male patients. Geographic disparities persist but have narrowed slightly. These findings highlight the importance of ongoing evaluation of access to TAR and the variability of surgical practices. Further studies are necessary to evaluate the continued development of TAR.

**Level of Evidence:**

Level III, retrospective cohort study.

## Graphical abstract

**Graphical Abstract:**
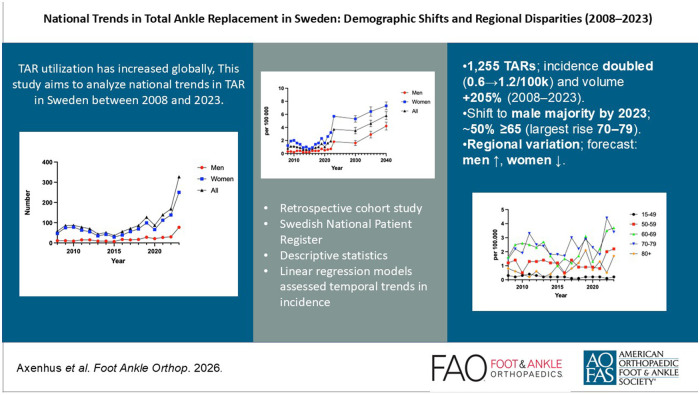

## Introduction

Total ankle replacement (TAR) is an established surgical option for patients with end-stage ankle arthritis, offering the potential to relieve pain while preserving joint mobility, in contrast to ankle arthrodesis, which eliminates motion at the joint.^1-3^ Over the past 2 decades, TAR has undergone significant developments in implant design, surgical technique, and perioperative management, contributing to improved survivorship and functional outcomes.^4-6^ Nevertheless, TAR is still performed far less frequently than hip or knee replacements and is often reserved for selected patient populations because of its technical complexity and a higher risk of complications compared with other joint arthroplasties.^6-8^

In Sweden, the use of TAR has been systematically recorded in national health care databases, including the Swedish National Board of Health and Welfare database and the Swedish Ankle Registry since 1997.^9^ These resources provide high-quality longitudinal data, enabling researchers to assess national trends and disparities over time. These databases are publicly accessible and available for use by researchers and health care professionals.

Although TAR utilization has increased globally, relatively little is known about how demographic factors such as age, sex, and regional differences affect access to this procedure within Sweden.^8^ Studying these disparities is essential for promoting equitable access to care and understanding whether certain patient groups are underrepresented or underserved.^10,11^ By examining the period from 2008 to 2023, this study aims to identify demographic trends in TAR and assess how evolution and utilization have been across the Swedish population.

## Methods

### Ethical Considerations

The data used in this study is sourced from publicly accessible, anonymized data, ensuring that individual identities are protected. As a result, the study does not require ethical approval or informed consent from participants. Additionally, because this is not based on a specific clinical trial, a clinical trial number is not applicable.

### Study Setting

Sweden provides universal health care through a system designed to ensure equal access for all residents. Emergency care, hospital treatments, and outpatient services are heavily subsidized, making health care widely available. Each resident is issued a unique personal identification number that serves as a critical link to both public and private health care systems and is recorded in national health registries for administrative purposes.

Sweden is divided into 21 regions, each responsible for overseeing health care delivery, public transportation, and regional school planning. These regions are managed by elected councils, which play a central role in ensuring that essential public services, including health care, are effectively provided to the population.

### Data Collection

This study is designed as a retrospective cohort analysis, using data obtained from the National Patient Register (NPR), maintained by the Swedish National Board of Health and Welfare.^9^ The NPR is a well-established and comprehensive data source, validated for use in epidemiologic research and retrospective studies.^12^ It includes extensive records on patient diagnoses, treatments, and health care utilization, making it a very useful tool for population-based studies.

The NPR is structured using the *International Statistical Classification of Diseases and Related Health Problems, 10th Revision* (*ICD-10*), to classify medical diagnoses and conditions.^13^ In addition to this, it consists of the corresponding Classification of Healthcare Procedures (CHP) codes to document surgical and medical interventions. Each procedure is given a specific alphanumeric code that allows for systematic collection, retrieval, and analysis of data. These standardized coding systems ensure consistency and accuracy in the reporting and retrieval of health care data, allowing for robust and reproducible analyses in research contexts.

### Patients

All individuals aged ≥15 years at the time of surgery who underwent a TAR and were recorded in the NPR from January 1, 2008, to December 31, 2023, were considered for inclusion. Only persons with a Swedish personal identification number were included. All TAR procedures were identified using NOMESCO surgical codes, which are part of the broader Swedish CHP (Code: NHB20).^14^ All surgeries with primary TAR without cement were included because of its overwhelmingly dominant number. Henceforth, TAR will refer to primary TAR without cement.

### Data Analysis

Descriptive statistics were applied, and results are presented as absolute numbers and percentages. To avoid duplicate counting, each unique personal identification number was included only once per calendar year, surgical procedure, and geographic region. Analyses were framed as exploratory. Annual procedure counts and incidence per 100 000 inhabitants were summarized with 95% CIs. Linear regression models assessed temporal trends in incidence with year as the independent variable, fitted separately for men and women (presented as slopes [β] and 95% CIs). Forecasts for 2024-2040 were generated from models fitted to 2015-2023 data and are presented as point estimates (with 95% prediction intervals where indicated). Analyses were conducted in R, version 4.5.2 (stats), and Python, 3.11 (scikit-learn 1.4, pandas 2.2, matplotlib 3.8).

## Results

**Figure 1.**
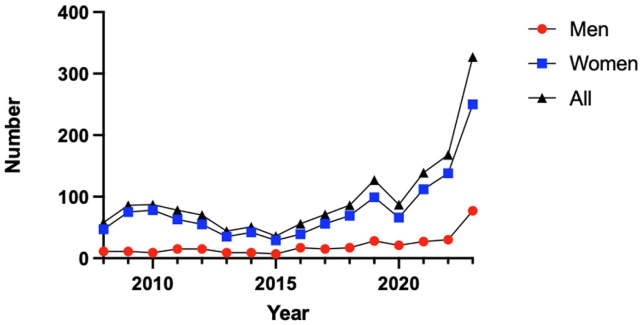
Total cases of total ankle replacement (TAR) in absolute numbers during 2008 to 2023.

In total 1255 patients had undergone TAR between 2008 and 2023. The majority were women (55%) and 47% of all ankle surgeries occurred in the age group 65+. Over the course of the 16-year study period, there was an increase of 205% with ankle replacement surgeries in absolute numbers from 63 cases in 2008 to 129 cases in 2023 (Table 1, Figure 1). There were 15 cemented TAR (Table 1).

**Table 1.** Absolute Numbers of Specific Procedures (2008-2023).

Operation	2008	2009	2010	2011	2012	2013	2014	2015	2016	2017	2018	2019	2020	2021	2022	2023
NHB09 Partial prosthesis without cement	2	0	4	0	6	14	3	3	4	1	1	2	0	0	0	0
NHB19 Partial prosthesis with cement	2	1	0	3	0	1	0	1	1	2	0	0	0	0	0	0
NHB20 Total prosthesis without cement	59	71	62	84	75	77	59	52	48	63	63	96	61	67	120	126
NHB30 Total prosthesis, hybrid technique	0	0	0	0	1	0	2	0	0	0	0	0	0	5	4	3

**Figure 2.**
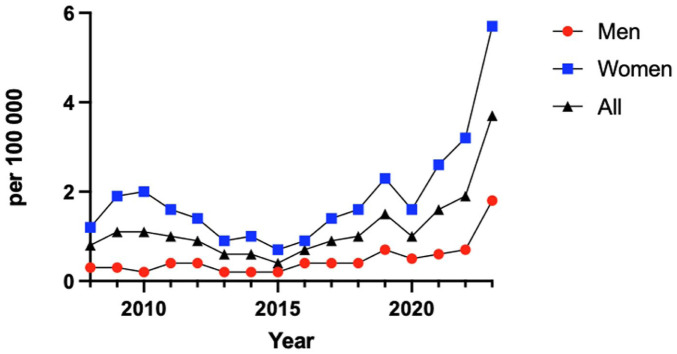
Total ankle replacement (TAR) procedures per 100 000 inhabitants per sex, 2008 to 2023.

The Swedish population increased by approximately 14% during the study period, predominantly because of an increase of the elderly population. Despite this, the incidence increased by 100% from 0.6 in 2008 to 1.2 per 100 000 inhabitants in 2023 (Figure 2).

**Figure 3.**
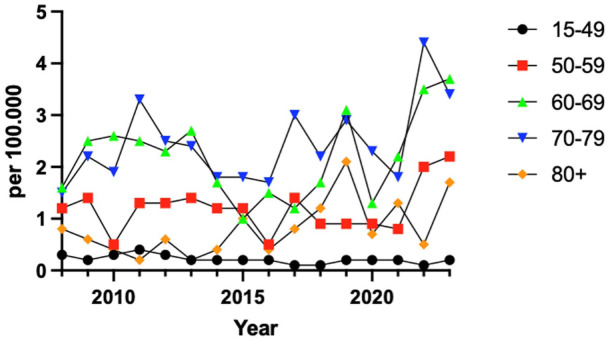
Total ankle replacement (TAR) incidence per 100 000 inhabitants per age group, 2008 to 2023.

The 60-69-year age group was already the most prevalent in 2008 with 17 cases and maintains this position in 2023 with 42 cases. The age group that has increased the most in relative terms is the 70-79-year group, from 10 cases in 2008 to 35 cases in 2023. The smallest change has occurred in the 15-49- and ≥80-year age groups (Figure 3).

The majority of all surgeries were female patients in 2008 with a total number of 38 cases compared to male patients with 22 cases. The male patient group increased from 2014 onward, surpassing the female patient group in 2019 and becoming the majority group by 2023, with a total of 70 cases.

**Figure 4.**
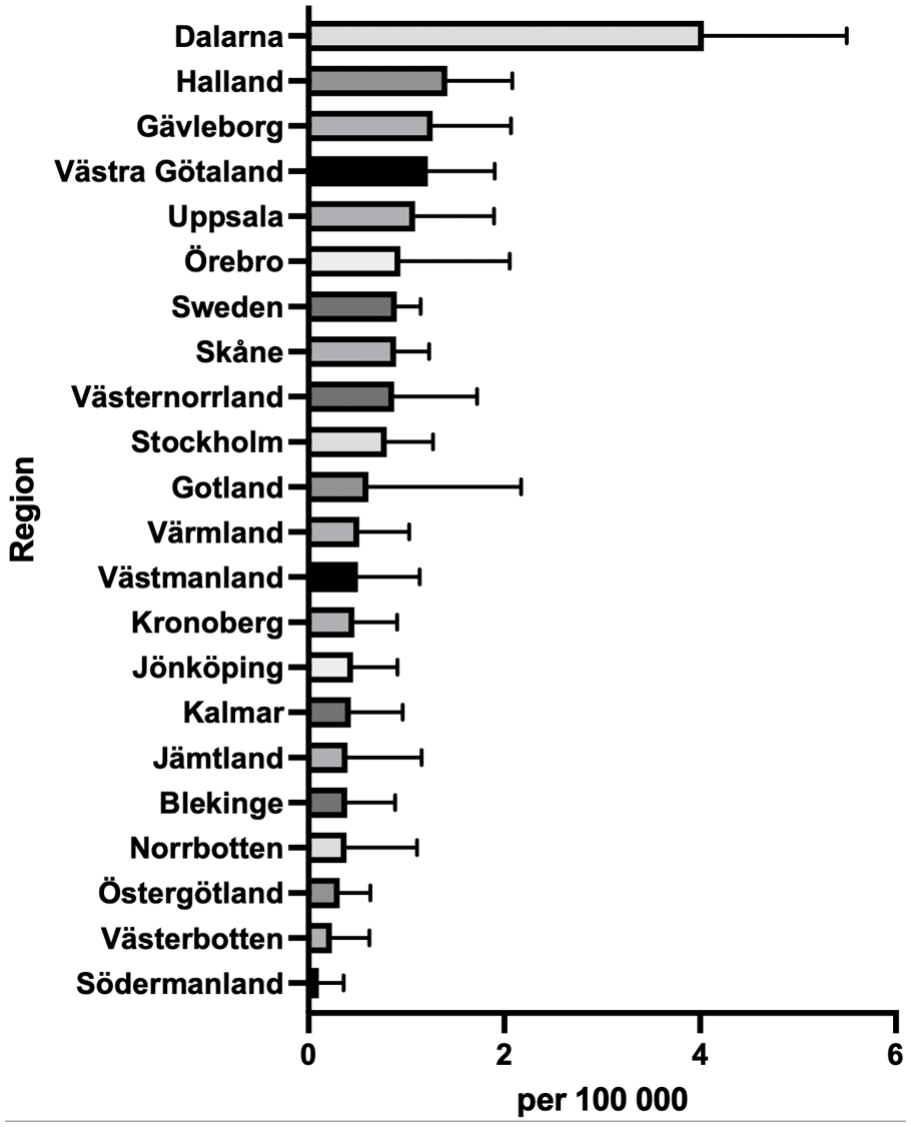
Mean regional incidence of total ankle replacement (TAR) procedures in 2008 to 2023.

The incidence of TAR differs markedly across Sweden during 2008-2023. Region Dalarna (population ~286 000) had the highest incidence in 2023, performing 3.9 surgeries per 100 000 inhabitants. Region Södermanland (population ~300 000) had the lowest incidence of 0.1 per 100 000 inhabitants (Figure 4).

**Figure 5.**
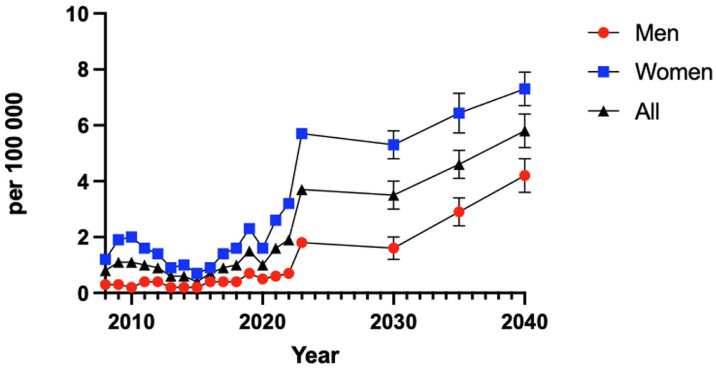
Predicative analysis of TAR incidence per sex until 2040. Error bars indicate 95% confidence interval.

Linear regression analysis showed a positive trend in incidence rates for men and a slight negative trend for women up until 2040 (Figure 5).

## Discussion

This study presents updated trends in TAR procedures in Sweden between 2008 and 2023. A steady increase in both the absolute number and incidence of TAR procedures was observed over the 16-year period, with a substantial rise in total cases of TAR and a doubling of the incidence rate per capita. Although the Swedish population grew by approximately 14% during the same period, primarily because of an aging demographic, this modest increase does not fully explain the substantial rise in TAR utilization.

There are 2 distinct peaks in the number of TAR during the study period. The first peak occurred in 2011 with a total of 84 cases, and the second in 2019 with 96 cases. This is in relation to the average of 67 cases per year throughout the study period. It is difficult to speculate on the causes of these 2 peaks. Moreover, the number of procedures doubled from 2021 to 2022. This trend continued in 2023, suggesting a sustained increase rather than a short-term fluctuation. Improved reporting alone is considered an unlikely explanation for this increase, as reporting has already been near optimal, with up to 100% reporting rates from the limited number of clinics performing TAR. Another possible explanation for the rise could be the introduction and use of the Infinity prosthesis, a fourth-generation TAR, introduced in Sweden in 2020. TAR has undergone substantial design evolution over the past decades, with systems aiming to improve alignment, fixation, and soft-tissue balancing while mitigating common failure mechanisms observed in earlier generations. Recent studies describe a continued transition toward modern implants as they have been found to have better survival.^3,11,15^ In parallel with implant and instrumentation advances, patient selection paradigms have evolved. Several factors historically considered absolute contraindications (eg, higher body mass index or diabetes) are increasingly treated as relative risk modifiers emphasizing individualized decision making.^16^ Over the study period, indications have broadened as implant designs advanced, surgeons are now more willing to consider TAR in younger or more active patients than before.^17^

Furthermore, the COVID-19 pandemic is believed to have had a negative impact, leading to postponed or cancelled procedures, which could be an explanation for the dip in 2020 and 2021; such fluctuations have been described elsewhere.^18,19^ When analyzing the trend without these two years there seem to be a steady sustained rise in TAR procedures since 2016. For the first time during the study period, 2 consecutive years with a high number of cases have been observed. The increase in TAR procedures from 2022 onward is likely multifactorial, driven by post-pandemic recovery, the adoption of newer implants, and rising patient demand for motion-preserving treatments.

The observed demographic shift in TAR patients is consistent with global findings, where procedures are increasingly performed in older populations.^8^ In this study, nearly half of the procedures occurred in patients aged 65 years and older. The most notable relative increase occurred in the 70-79 age group, which grew by 350%, suggesting a growing acceptance of TAR in elderly patients. Despite concerns regarding longevity and comorbidities in this age group, studies have shown promising functional outcomes and implant survivorship in older patients.^7,20-22^

This study focused on cementless TAR procedures because of their clear predominance in national data. Cemented TAR was exceedingly uncommon with only 15 cases, mostly in the last 3 years. This recent appearance of a few cemented implants, although limited, suggests that cemented fixation is occasionally being explored. This approach aligns with international trends favoring cementless fixation, which has been associated with favorable clinical outcomes and lower revision rates. The initial predominance of female patients aligns with previous registry data.^23,24^ However, by 2023, this distribution had reversed, with male patients comprising the majority. This shift may reflect changes in physical activity levels, health care–seeking behavior, or evolving surgical indications. Further investigation into sex-specific factors influencing TAR utilization would be valuable.

The observed shift in regional incidence between 2008 and 2023 is likely attributable to an increase in TAR procedures in previously low-volume regions, rather than a decline in procedure rates in the leading regions from 2008. The results suggest persistent differences in TAR access and utilization across regions, potentially driven by unequal distribution of surgical expertise, inconsistent referral patterns, and limited availability of specialized centers.^10,25^ The future predictive model indicated an increase for men and a decrease for women. However, these projections should be interpreted cautiously. Linear models assume that past trends continue unchanged, which may not hold true in practice. Future changes in diagnostic criteria, reporting systems, or prevention measures could significantly alter these predictions.

Disparities in TAR utilization by age seem to diminish with increased indications in recent years. Regarding sex, it may be surprising that men have surpassed women in TAR, given that existing literature reports a higher prevalence of joint disease in women due to rheumatic conditions, hormonal factors, and longer life expectancy.^26-28^ Better treatments for rheumatic diseases could be a negative driver.^29^ Men might suffer from more post-traumatic arthritis in the ankle.^30,31^ While there remain disparities in TAR utilization by region, sex, and age, our data do not allow determination of whether specific patient groups were truly under-served. Addressing this question would require additional data and is an important area for future research.

This study is based on registry data, and although the NPR is known for high coverage and accuracy, certain clinical variables, such as patient comorbidities, implant types, surgical technique, and functional outcomes, are not available. Crude incidence rates were reported rather than age-standardized rates. Given that Sweden’s population has gradually aged over the study period, crude rates may underestimate the increase in utilization to some extent. Moreover, projections for future TAR incidence are exploratory and should be interpreted with caution as they do not account for non-linear trends (COVID-19 or changes due to new implants). The data set did not include case-level *ICD-10* diagnostic codes, limiting the ability to determine whether the observed trends reflect changes in surgical utilization or shifts in disease epidemiology. Despite these limitations, the use of a comprehensive national database provides robust insights into temporal and regional trends in TAR procedures across Sweden.

## Conclusion

TAR procedures have markedly increased in Sweden from 2008 to 2023, with a growing proportion performed in older patients and a shift in gender predominance. The widening regional adoption of TAR suggests increased acceptance and accessibility, although variability remains. These findings highlight the evolving role of TAR in modern orthopaedic practice.

## Supplemental Material

sj-pdf-1-fao-10.1177_24730114261423178 – Supplemental material for National Trends in Total Ankle Replacement in Sweden: Demographic Shifts and Regional Disparities (2008-2023) Supplemental material, sj-pdf-1-fao-10.1177_24730114261423178 for National Trends in Total Ankle Replacement in Sweden: Demographic Shifts and Regional Disparities (2008-2023) by Michael Axenhus, Fatih Uludag and Viktor Mili-Schmidt in Foot & Ankle Orthopaedics
